# Case Report: Late-Onset Autosomal Recessive Cerebellar Ataxia Associated With *SYNE1* Mutation in a Chinese Family

**DOI:** 10.3389/fgene.2022.795188

**Published:** 2022-02-23

**Authors:** Nannan Qian, Taohua Wei, Wenming Yang, Jiuxiang Wang, Shijie Zhang, Shan Jin, Wei Dong, Wenjie Hao, Yue Yang, Ru Huang

**Affiliations:** ^1^ Graduate School, Anhui University of Traditional Chinese Medicine, Hefei, China; ^2^ The First Affiliated Hospital of Anhui University of Traditional Chinese Medicine, Hefei, China; ^3^ Key Laboratory of Xin’an Medicine Ministry of Education, Hefei, China; ^4^ V-Medical Laboratory Co., Ltd, Hangzhou, China

**Keywords:** case report, autosomal recessive cerebellar ataxia, ARCA-1, SYNE1 ataxia, SYNE1 gene, SCAR8

## Abstract

Autosomal recessive cerebellar ataxia type 1 (ARCA-1), also known as autosomal recessive spinocerebellar ataxia type 8 (SCAR8), is caused by spectrin repeat containing nuclear envelope protein 1 (*SYNE1*) gene mutation. Nesprin-1, encoded by *SYNE1*, is widely expressed in various tissues, especially in the striated muscle and cerebellum. The destruction of Nesprin-1 is related to neuronal and neuromuscular lesions. It has been reported that *SYNE1* gene variation is associated with Emery-Dreifuss muscular dystrophy type 4, arthrogryposis multiplex congenita, SCAR8, and dilated cardiomyopathy. The clinical manifestations of SCAR8 are mainly characterized by relatively pure cerebellar ataxia and may be accompanied by upper and/or lower motor neuron dysfunction. Some affected people may also display cerebellar cognitive affective syndrome. It is conventionally held that the age at the onset of SCAR8 is between 6 and 42 years (the median age is 17 years). Here, we report a pedigree with SCAR8 where the onset age in the proband is 48 years. This case report extends the genetic profile and clinical features of SCAR8. A new pathogenic site (c.7578del; p.S2526Sfs*8) located in *SYNE1*, which is the genetic cause of the patient, was identified via whole exome sequencing (WES).

## Introduction

Hereditary ataxia is a group of highly heterogeneous genetic degenerative diseases characterized by chronic progressive ataxia. Its phenotype includs ataxic gait and uncoordinated eye movement, language and hand movement, and often involves pathological changes mainly affecting the spinal cord, cerebellum, and brainstem (Jayadev and Bird, 2013). Hereditary ataxia includes autosomal dominant ataxia, autosomal recessive ataxia, X-linked ataxia, and ataxia associated with mitochondrial diseases (Braga Neto et al., 2016). SCAR8 is a type of autosomal recessive ataxia, which is clinically characterized by mild cerebellar ataxia and may be accompanied by upper and/or lower motor neuron dysfunction. Some people may also experience cerebellar cognitive affective syndrome (Laforce et al., 2010). It is reported that the incidence of autosomal recessive ataxia is 0–7.2/100,000 (Ruano et al., 2014). Friedreich ataxia and ataxia-telangiectasia are the most common autosomal recessive ataxia, while other types such as SCAR8 are less common (Palau and Espinós, 2006). *SYNE1*, the gene responsible for SCAR8 first reported in French-Canadian patients in Quebec, Canada (Gros-Louis et al., 2007), was considered to be limited to a specific section of the French-Canadian population in a long time (Synofzik et al., 2019). With the development of high-throughput sequencing technology, genetic variations in *SYNE1* have been reported worldwide (Gama et al., 2016; Algahtani et al., 2017; Peng et al., 2018; Swan et al., 2018; Arias et al., 2019; Duan et al., 2021). According to a multicenter study of *SYNE1* mutations (Mademan et al., 2016), the age of onset is between 6 and 42 years (the median age is 17 years). Not all patients show the classical *SYNE1* phenotype of pure cerebellar ataxia, but all exhibit ataxia and motor neuron disease, which involves upper and/or lower motor neuron dysfunction. Here, we report a Chinese SCAR8 patient carrying a new homozygous mutation (c.7578del; p.S2526Sfs*8) in *SYNE1*, which has just been observed for the first time. The patient showed late-onset at the age of 48, and the clinical symptoms were characterized by slowly progressive cerebellar ataxia accompanied by motor neuron dysfunction.

## Methods

This study was approved by the Ethics Committee of The First Affiliated Hospital of Anhui University of Chinese Medicine (batch number: 2021AH-60). The patient and her relatives were informed and provided written consent for the study and the publication of this report.

### Case Description

**FIGURE 1:**
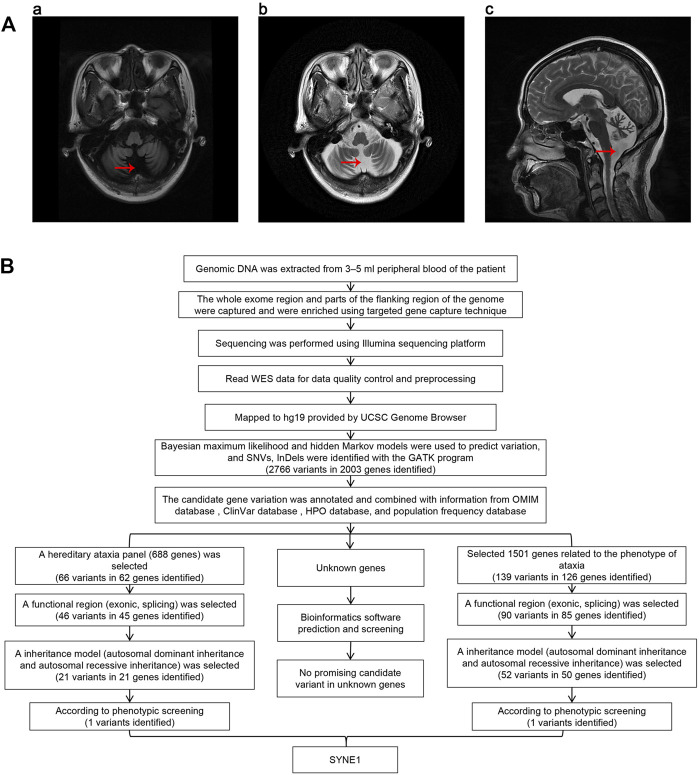
**(A)** Brain MRI of proband. The red arrows in the charts of “a,” “b,” and “c” show that the sulcus in both cerebellar hemispheres is significantly increased and widened, indicating cerebellar atrophy. **(B)** The workflow of analysis of whole exome sequencing data.

At the age of 48 years, a female patient began to experience unsteady gait and trouble walking up stairs after prolonged standing. The patient did not pay attention to the symptoms at the time and did not seek medical treatment. At the age of 53 years, the patient found herself a symptom of slurred speech. After 1 year she went to the local hospital where on admission CT showed cerebellar atrophy. The cause was not clear, so the patient was discharged after symptomatic treatment consisting of oral butylphthalide soft capsules and pyridostigmine bromide tablets. Despite adhering to outpatient follow-up, the patient’s symptoms did not improve. The patient continued having difficulty with the stairs, dizziness after prolonged standing, and occasional falls, so she was admitted to our hospital for seeking treatment. On admission, the patient demonstrated an ataxic gait, poor distance discrimination, abnormal finger-to-nose test, dysphagia, slurred speech, and negative nystagmus. The physical exam of the patient showed that she had a decreased muscle tone in the lower extremities with weak tendon reflex, unstable heel-knee-shin test, and dysdiadochokinesia. Moreover, during the Romberg’s test, the patient was unstable in the stage of closing eyes and opening eyes. Her evaluation on the Scale for the Assessment and Rating of Ataxia (SARA) demonstrated 15 points out of a total of 40. In addition, she got 19/30 on the Mini Mental State Exam (MMSE) and 14/30 on the Montreal Cognitive Assessment (MoCA), respectively (the patient was illiterate). The patient self-reported no family history of neurological disease, and the brain MRI showed severe cerebellar atrophy (Figure 1A). Her father had succumbed to cerebral infarction at the age of 70 and her mother had died due to lung cancer at the age of 65. The patient stated that her parents had a non-consanguineous marriage, and neither of her parents showed symptoms of cerebellar ataxia. The patient has a 58-year-old brother and a 51-year-old sister. Neither of them showed symptoms of cerebellar ataxia, as examined by the chief physician. The patient’s 33-years-old daughter and two granddaughters (7 and 5 years old, respectively) showed no symptoms of cerebellar ataxia, as ascertained after examination by the chief physician. The patient was examined for short tandem repeat expansion in the responsible genes for SCA1, SCA2, SCA3, SCA6, SCA7, SCA8, SCA10, SCA12, SCA17, and DRPLA, which are the most common subtypes of spinocerebellar ataxia (SCA) in China. The results showed no abnormalities (Supplementary Figure S1).

### Whole-Exome Sequencing

Genomic DNA was extracted from 3–5 ml peripheral blood of the patient treated with the anticoagulant EDTA. All exonic regions and parts of their flanking intronic regions were captured and enriched by using Agilent SureSelect Human All Exon V6 Kit. The point mutations in exons, some flanking regions of selected genes, and insertion and deletion variations within 20 bp (small variation) were analyzed by using the Illumina sequencing platform (San Diego, California, United States). The average sequencing depth of this test was 210.56×, and the coverage rate of the target fragment reaching 20× was 97.74%. The Q30 score was greater than 90%. Data quality control and preprocessing were performed, followed by strict quality control of the sequencing data with removal of joint contamination and low-quality sequencing data to ensure the authenticity and reliability of the data. The high-quality sequencing data was compared with the human genome reference sequence (GRCh37/hg19) provided by the UCSC Genome Browser database (http://genome.ucsc.edu/) using the Burrows-Wheeler transform (BWT) algorithm. The coverage and the sequencing quality of the target region were evaluated at the same time. Bayesian maximum likelihood and hidden Markov models were used to predict variation, and single nucleotide variants (SNVs) and InDels (insertions and deletions) were identified using HaplotypeCalleralgorithm in GATK (http://www.broadinstitute.org/gsa/wiki/index.php/Home_Page). The candidate gene variants were annotated and combined with information from the Online Mendelian Inheritance in Man (OMIM) database (https://omim.org/), ClinVar database (https://www.ncbi.nlm.nih.gov/clinvar/), Human Phenotype Ontology (HPO) database (https://hpo.jax.org/app/), and population frequency database (http://www.allelefrequencies.net/). Next, we chose two methods to screen candidate genes. One way is to screen candidates in a selected gene panel related to hereditary ataxia including 688 known genes (Supplementary Table S1). First, we identified 66 variants in 62 genes within this panel. Then functional regions (exonic, splicing) were selected for a following screening, and candidates were narrowed down to 46 variants in 45 genes. In third step, we selected variants according to their inheritance patterns, such as autosomal dominant inheritance, autosomal recessive inheritance and X-linked inheritance, and 21 variants in 21 genes were kept after this round screening. Finally, according to the patient’s clinical phenotypes, *SYNE1* mutation was screened out among 21 variants. The other way is to screen 1,501 known genes related to the phenotype of ataxia in the databases (Supplementary Table S2). We could identify 139 variants in 126 genes after the first round screening. Then a functional regions (exonic, splicing) were selected for a following screening, and 90 variants in 85 genes were kept. In third round, we screened variants according to their inheritance patterns, candidates were narrowed down to 52 variants in 50 genes. Lastly, *SYNE1* mutation was picked out among 52 variants according to the patient’s clinical phenotypes. In addition, we performed bioinformatics analysis and prediction for unknown genes as well. We selected variants with absent or frequency <1% in all the population databases, including dbSNP, gnomAD, ExAC, g1000 and ESP6500, Exac_all, Exac_eas. We used bioinformatics prediction software (SIFT, Polyphen-2, Mutation Taster, LR pred, Ljb23_metasvm, REVEL, dbscSNVada and dbscSNV_rf) to predict the pathogenicity of variation. However, we did not find any promising candidate variant (Figure 1B). The mutation separation were verified among family members by Sanger sequencing.

## Results

**FIGURE 2:**
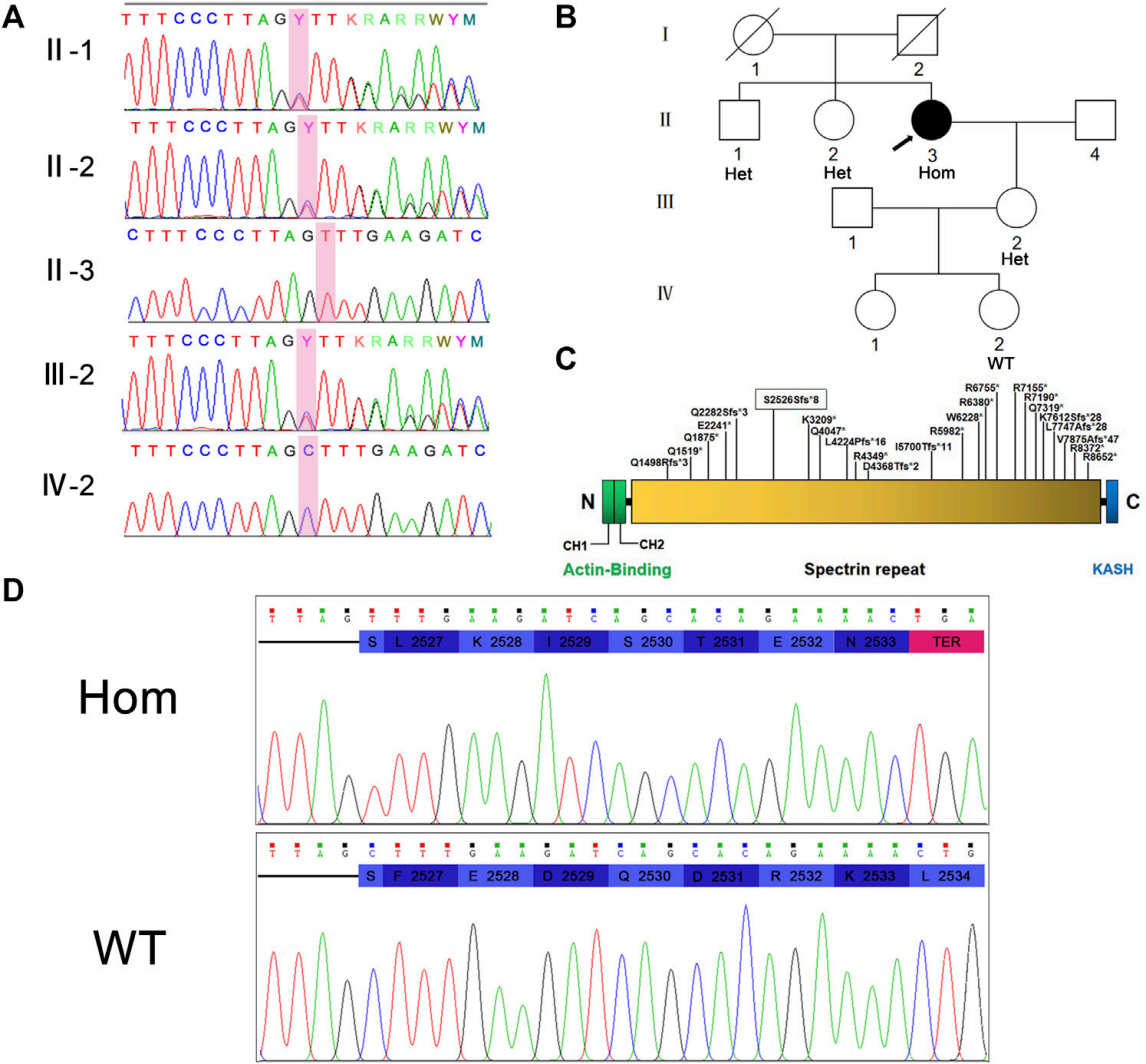
**(A)** Sanger sequencing peaks of family members. **(B)** Pedigree of family. II-3 is the proband. **(C)** Nesprin-1 and the location of previously reported truncated variants are illustrated. The variations found in this study are marked with boxes. **(D)** Translation pattern diagram. The mutation c.7578del (p.S2526Sfs*8) occurred in exon 51 of *SYNE1* gene, leaving the serine at position 2,526 unchanged. The subsequent codon frameshift mutation resulted in the suspension of translation of 7 amino acids and the deletion of protein fragments (including KASH domain).

The NM_033071:c.7578del (p.S2526Sfs*8) variant in *SYNE1* was spotted by two screening methods. The result of Sanger sequencing confirmed the patient is a homozygous carrier of NM_033071:c.7578del (p.S2526Sfs*8) variant. In order to further clarify the genetic status within the family, the available family members (the patient’s brother, sister, daughter, and one of granddaughters) were tested for *SYNE1* gene. The result showed that her brother, sister, and daughter carried one copy of the NM_033071:c.7578del variant in *SYNE1*, while her 5-year-old granddaughter (IV-2) did not have this variant (Figures 2A,B). Furthermore, no pathogenic variant was detected in responsible genes for SCA1, SCA2, SCA3, SCA6, SCA7, SCA8, SCA10, SCA12, SCA17 and DRPLA by filter screening using whole exome sequencing data in the patient (data not shown).

Hence, the mutation in exon 51 of *SYNE1* was considered to be the genetic cause for the patient. This mutation did not affect the coding of the serine at position 2,526 per se, but a frameshift mutation occurred and changed the reading of subsequent codons. This resulted in transcription and translation errors which, in turn, affected protein function (PVS1) (null variant, including nonsense, frameshift, canonical+/−1 or 2 splice sites, initiation codon, single or multi-exon deletion in a gene where loss of function (LOF) is a known mechanism of disease). This mutation is not included in many population databases including 1,000 Genomes database, ExAC database, gnomAD database, Esp6500 database, HGMD database, and ClinVar database. The mutation does not belong to polymorphic loci, and the frequency of occurrence in the population is very low (PM2) (absent from controls, or at extremely low frequency if recessive in Exome Sequencing Project, 1,000 Genomes or ExAC). Based on the above evidence, the variant was classified as a likely pathogenic (ACMG criteria PVS1 and PM2) (Richards et al., 2015). The pathogenicity of *SYNE1* gene mutation was predicted by using the online bioinformatics software LR pred, Mutation Taster, and Ljb23_metasvm, and the results revealed it as a disease-causing mutation.

## Discussion

*SYNE1*, also known as nuclear envelope spectrin 1 (Nesprin-1), which is located at 6q25.2, is one of the largest genes in the human genome. It has 146 exons, encoding 27,652 base pairs of messenger RNA, translated into a protein of 8,797 amino acids, Nesprin-1. Nesprin-1 is widely expressed in various tissues, especially in striated muscles and the cerebellum (King et al., 2014). Nesprin-1 is located on the outer nuclear membrane and binds to F-actin through the N-terminal. Through the association between its C-terminal (Klarsicht-ANC-Syne-homology) KASH domain and the SUN domain of the perinuclear space SUN1/2, the linker of nucleoskeleton and cytoskeleton (LINC) complex is formed. The LINC complex connects the nuclear membrane to the cytoskeleton elements, which include actin filaments and actin microtubule networks (Nikolova-Krstevski et al., 2011). If the *SYNE1* gene is mutated, the structure of Nesprin-1 protein will be changed, and in turn the stability, size, and shape of nucleus will also be affected. Thus the defected composition of the nuclear layer and the nuclear membranes will result in various degenerative diseases which affect the development of striated muscles, peripheral nerves, bones and fat, as well as premature aging syndrome (Sur et al., 2014). *Drosophila* larvae with the deletion of KASH domain exhibits motor disturbance of myasthenia, which proves the importance of KASH in structural dyskinesia phenotype. Further experiments showed that abnormal neuromuscular transmission and postsynaptic imbalance of glutamate receptors were observed at the neuromuscular junctions in *Drosophila* larvae with the deletion of KASH, and the percentage of receptors containing glutamate receptor type A (GluRIIA) decreased. The dyskinesia phenotype of KASH deletion was prevented by GluRIIA overexpression, which indicated that the motor disturbance associated with KASH domain deletion was caused by the decrease in GluRIIA expression (Morel et al., 2014).

It has been confirmed that *SYNE1* gene variation is associated with Emery-Dreifuss muscular dystrophy type 4 (EDMD4) (OMIM#612998), myogenic-type arthrogryposis multiplex congenita-3 (AMC3) (OMIM#618484), and SCAR8 (OMIM#610743) (Gama et al., 2016; Indelicato et al., 2019; Noreau et al., 2013). In addition, it has been reported that *SYNE1* gene mutation may be associated with dilated cardiomyopathy (DCM) (Zhao et al., 2021). Nesprin-1 binds emerin and lamins A/C to form a network in muscles, connecting nucleoskeleton to inner nuclear membrane (INM), outer nuclear membrane (ONM), membrane organelle, sarcomere, and actin to form cytoskeleton (Warren et al., 2005). In addition, the functional and structural abnormalities of nesprin/lamin/emerin interaction may play a role in the muscle-specific pathogenesis of Emery-Dreifuss muscular dystrophy type 4 (Zhang et al., 2007). The C-terminal heterozygous missense variant of *SYNE1* gene has been identified to be associated with sporadic dilated cardiomyopathy and Emery-Dreifuss muscular dystrophy type 4, and a patient with C-terminal truncation mutation of *SYNE1* gene reportedly developed cardiomyopathy (Indelicato et al., 2019). Three novel C-terminal missense mutations were identified in 218 patients with sporadic dilated cardiomyopathy. The expression of these mutants disrupted the Nesprin-1/lamin/SUN interaction and led to nuclear morphological defects (Zhou et al., 2017). In another report, a nonsense homozygous mutation at the C-terminal in a patient with AMC was identified, resulting in truncation of the KASH domain. Interestingly, mRNA analysis showed that the transcript of the mutant was expressed at the same level of wild type (Baumann et al., 2017). We found that the truncation caused by previously reported *SYNE1* mutations near the N-terminal of Nesprin-1 resulted in late-onset, while those near the C-terminal of Nesprin-1 resulted in early-onset (Figure 2C, Supplementary Figure S2, Supplementary Table S1). Nesprin-1 has multiple internal promoters which can produce shorter isoforms with a common C-terminal region, but truncated N-terminal, such as Nesprin-1-giant, Nesprin-1α2, Nesprin-1α1, Nesprin-1β2, Nesprin-1γ, p53 KASH^Nesp1^, and p53 KASH^Nesp1^ (Rajgor et al., 2012; Baumann et al., 2017). Some studies have shown that the dysfunction of a KASH-LESS Nesprin1 giant (KLNes1g) is the basis for pathogenesis of SCAR8, and it has been confirmed that KLNes1g is related to the function of vesicles and dendritic membrane (Razafsky and Hodzic, 2015). There are different distribution patterns of Nesprin-1 isoforms in INM and ONM. Based on the points above, we hypothesized when the truncation is close to the N-terminal of Nesprin-1, the influence of truncation mutation to the Nesprin-1 isoforms is less or it only affects the function of Nesprin-1giant and KLNes1g. Furthermore, the structural homologue Nesprin-2-giant has a certain compensation effect on the function of Nesprin-1giant (Razafsky and Hodzic, 2015); hence, the onset caused by their defect is later. On the contrary, when the truncation was close to the C-terminal of Nesprin-1, the mutation not only affected the function of Nesprin-1giant and KLNes1g, but also the function of all other Nesprin-1 isoforms with early-onset. Therefore, the hypothesis needs to be further studied. The short α isoforms of Nesprin, Nesprin-1α2 and Nesprin-2α2, are almost exclusively expressed in the heart and skeletal muscle (Apel et al., 2000). EDMD showed only cardiac and skeletal muscle involvement. All known Nesprin mutations associated with EDMD or DCM are located in the α isoform sequence (Duong et al., 2014). The tissue-specific distribution of α isoforms and the location of pathogenic *SYNE1* mutations may indicate that α isoforms have some specific functions that are not available in the full-length “giant” form. The loss of this function may be the cause of EDMD and DCM. Furthermore, it can be inferred that the tissue-specific distribution of α isoform and the location of pathogenic *SYNE1* mutation may be the reasons for the pleiotropy of *SYNE1* gene mutation. However, further research is needed to address this issue in the future. The frameshift mutation c.7578del (p.S2526Sfs*8) of the *SYNE1* gene we reported results in an early interruption in the process of Nesprin-1 translation. However, it may also lead to mRNA degradation through the nonsense-mediated mRNA decay (NMD) pathway (Kölbel et al., 2019), resulting in the inability of protein translation. Both mechanisms need to be verified via biological experiments in the future.

The typical manifestation of SCAR8 includes cerebellar syndromes, such as cerebellar ataxia, dysarthria, dyslexia, saccade, etc. Other features include upper motor neuron dysfunction (spasm, hyperreflexia, Babinski sign) and/or lower motor neuron dysfunction (muscular atrophy, impaired reflex, muscle bundle tremor). Some people may display cerebellar cognitive affective syndrome, that is, obvious attention deficit. Executive function, language working memory, and visual space/visual construction skills may also be affected (Beaudin et al., 1993; Algahtani et al., 2017). In this work, we report that the *SYNE1* gene with a mutation of c.7578del (p.S2526Sfs*8) in exon 51 has an early-translation interruption in the Nesprin-1 translation process, resulting in a deletion of protein fragment including the KASH domain (Figure 2D). It was previously reported that the onset age of SCAR8 ranges from 6 to 42 years, and the median age is 17 years. The proband in this family displayed a late-onset slowly progressive cerebellar ataxia with dysfunction of lower motor neurons, had a 7-years history of progression, cerebellar atrophy, which were caused by carrying the homozygous *SYNE1* mutation of NM_033071:c.7578del (p.S2526Sfs*8). It is interesting to note that there was no consanguineous marriage between the parents of the proband. But they came from the same geographical region, which indicates that they may originate from a common ancestor. However, uniparental disomy (UPD) cannot be ruled out as a possible mechanism. As the whole exome sequencing data of the proband is available, this could be a new hypothesis to investigate in the future. The proband’s brother and sister are heterozygous carriers and neither of them nor the deceased parents developed the same disease. Therefore, carrying the homozygous *SYNE1* mutation is the main genetic cause of SCAR8 for this patient.

At present, the effective drug treatment in degenerative cerebellar diseases is very limited, and the treatment is confined to only specific type of diseases and symptoms (Ilg et al., 2014). Physiotherapy is the main treatment of ataxia currently (Synofzik and Ilg, 2014). The characteristics of ataxia gait include difficulties with inter- and intra-limb coordination, a slow walking speed, irregular steps and reduced postural stability (Ioffe et al., 2007). Studies have shown that a series of physiotherapy interventions have a positive effect on ataxia (Cassidy et al., 2018). The difficulty of inter- and intra-limb coordination is one of the main manifestations of cerebellar ataxia. Gait coordination is related to the function of paleocerbellum and neocerebellum. Gait coordination can be improved through exercises to improve postural stability, reduction of degrees of freedom (such as positioning, splinting), and providing external support (such as orthotics, walking aids) (Delatycki et al., 2005; Ilg et al., 2010; Serrao et al., 2017). In one study, investigators assessed the long-term effects of a 4-weeks intensive coordination training and subsequent 1-year continuation of training at home. The results showed that there was a trend towards lower SARA scores in patients with 4 weeks of training and 1 year of training compared to pre training. And patients walked longer distances unaided (Ilg et al., 2010). Patients with ataxia may experience reduced ability of adapting to environmental changes. The ability of adapting to environmental changes is mainly controlled by the neocerebellum. The ability of adapting to environmental changes can be improved through adaptive gait training. A study showed that 10 patients with degenerative cerebellar ataxia were trained in gait adaptation. Visual step targets and obstacles are projected on the surface of the treadmill running belt. After 10 times of training within 5 weeks (1 h per time), the success rate of patients avoiding obstacles has increased significantly (Fonteyn et al., 2014a). The motor learning ability of patients with cerebellar injury will also be impaired. Motor learning abilities were related to the neocerebellum. Stepwise prompts to learn and high levels of repetition exercises should be used (Kelly and Shanley, 2016). When patients experience a decrease in consolidation motor skills, they need to practice the necessary skills with high intensity and pay attention to walking exercises consciously (Im et al., 2017). These training sessions primarily improved neocerebellum function. Patients with reduced posture control and muscle tone can be given special strength and balance training as well as compensatory strategies to increase stability (Kelly and Shanley, 2016). These training sessions primarily improved paleocerebellum function. Physiotherapy usually combines balance, gait, coordination, strength, endurance and posture (Fonteyn et al., 2014b). Often, the combination of multiple training methods can improve ataxia (Miyai et al., 2012; Schroeder, 2013). Recently, several commercial video training games have been used to train patients with ataxia. Commercial video training games can provide a novel and beneficial treatment tool (Ilg et al., 2012; Synofzik et al., 2013; Schatton et al., 2017). In addition, exercises like *Tai Chi* and Chinese acupuncture treatment may have potential benefits for patients with ataxia (Winser et al., 2018; Wang et al., 2021).

Since the occurrence of *SYNE1*-related SCAR8 cases in Canada (Gros-Louis et al., 2007), up to September 2021, a total of 229 *SYNE1* gene mutations have been reported according to the professional edition of HGMD database (Stenson et al., 2020). The frameshift mutation c.7578del (p.S2526Sfs*8) we reported is not mentioned in this database, and hence, is a new type of mutation. The symptoms of the patient reported here are similar to those of typical cerebellar ataxia previously reported, and are additionally accompanied by lower motor neuron dysfunction. More importantly, the onset age of this patient exceeds the onset age range previously reported, and our report expands the clinical-pathological features and gene profile in SCAR8.

## Data Availability

The datasets for this article are not publicly available due to concerns regarding participant/patient anonymity. Requests to access the datasets should be directed to the corresponding author.
