# Supplementary material for: Case Report: Late-Onset Autosomal Recessive Cerebellar Ataxia Associated With SYNE1 Mutation in a Chinese Family
Source: Front Genet. 2022 Feb 23;13:795188. doi: 10.3389/fgene.2022.795188 (PMC8905644; doi:10.3389/fgene.2022.795188)
Supplement: Supplementary file 3 [file DataSheet1.docx]

Supplementary Material


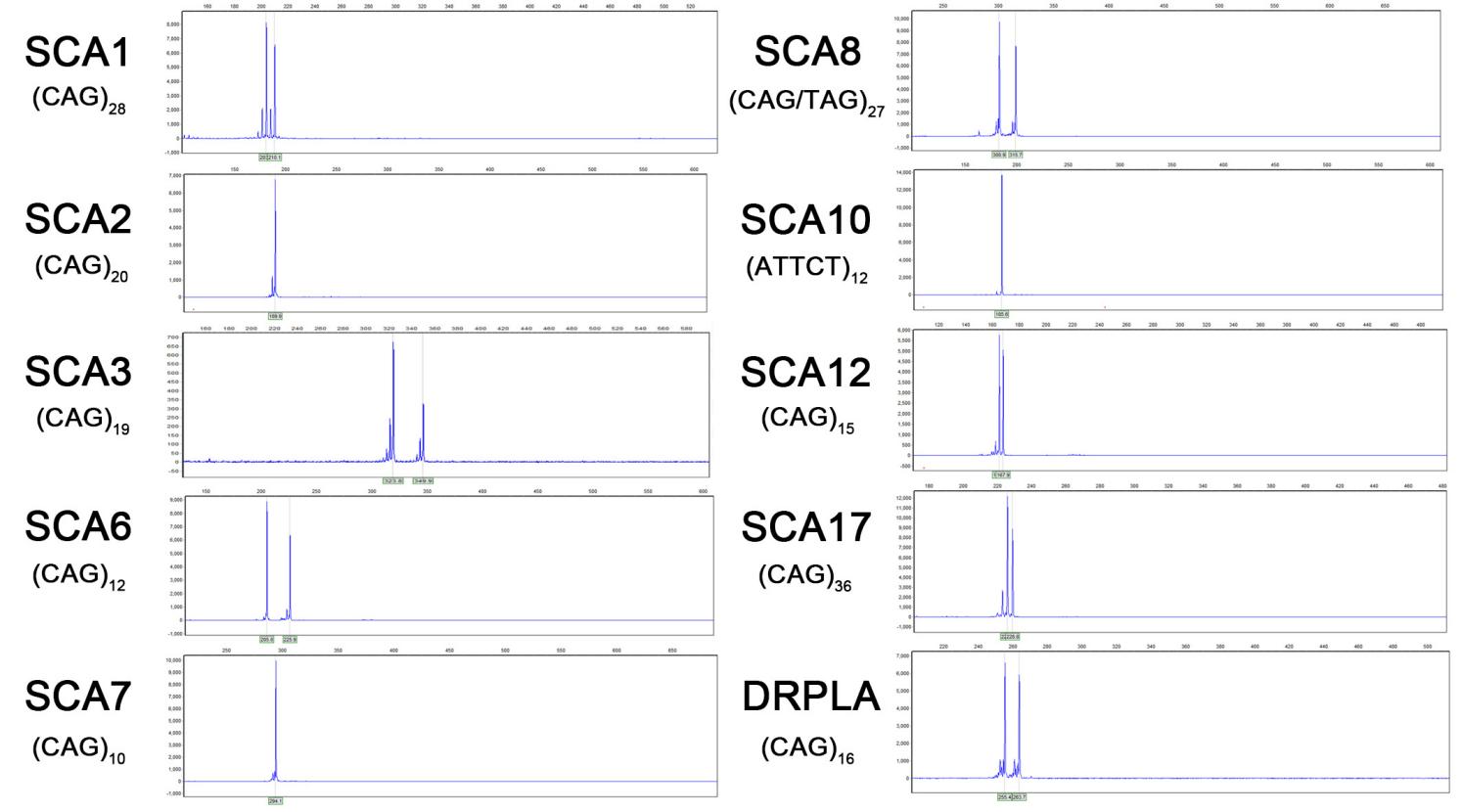


**Supplementary Figure S1.** PCR and capillary electrophoresis were used to detect the dynamic mutation of pathogenic genes of SCA1, SCA2, SCA3, SCA6, SCA7, SCA8, SCA10, SCA12, SCA17 and DRPLA.


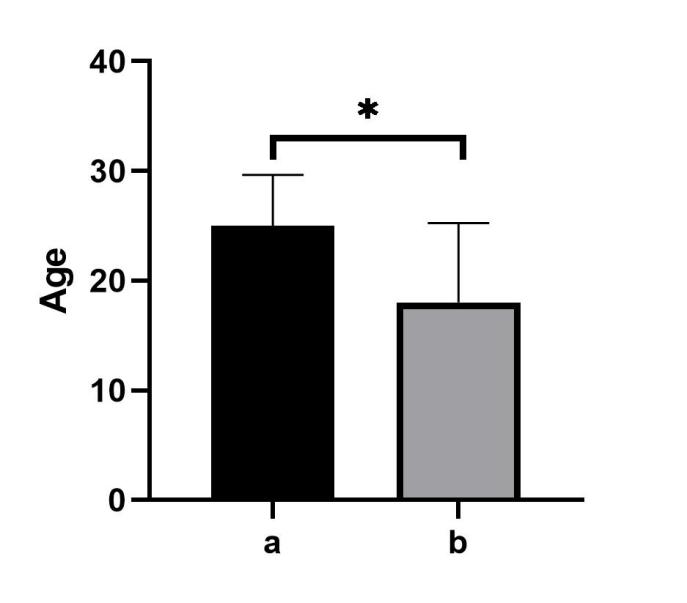


**Supplementary Figure S2.** Comparative Study on the age of onset in groups with different positions of truncated variants.The truncated variants of "a" group and "b" group were located in the interval [1, 4398] and [4399, 8797] respectively. There was significant difference in the age of onset between the two groups (P < 0.05).

**Supplementary Table S1.** Mutations in the *SYNE1* gene that have previously been reported to cause Nesprin-1 truncation and age of onset.

| Variant Name | Amino Acid Change | Age of Onset | Reference |
| --- | --- | --- | --- |
| c.4491delT | p.Q1498Rfs*3 | 24 | (Duan et al., 2021) |
| c.4555C>T | p.Q1519* | 33 | (Yoshinaga et al., 2017) |
| c.5623C>T | p.Q1875* | 26 | (Indelicato et al., 2019) |
| c.6721G>T | p.E2241* | 27 | (Indelicato et al., 2019) |
| c.6843del | p.Q2282Sfs*3 | 22 | (Yoshinaga et al., 2017) |
| c.9625A>T | p.K3209* | 30 | (Valentina Castillo et al., 2021) |
| c.12139C>T | p.Q4047* | 19 | (Holla et al., 2021) |
| c.12670dupC | p.L4224Pfs*16 | 23 | (Duan et al., 2021) |
| c.13045C>T | p.R4349* | 27 | (Arias et al., 2019) |
| c.13102delG | p.D4368Tfs*2 | 27 | (Duan et al., 2021) |
| c.17099-17100del | p.I5700Tfs*11 | 33 | (Arias et al., 2019) |
| c.17944C>T | p.R5982* | 15 | (Peng et al., 2018) |
| c.18684G>A | p.W6228* | 15 | (Peng et al., 2018) |
| c.19138C>T | p.R6380* | 11 | (Kume et al., 2019) |
| c.21463C>T | p.R7155* | 15 | (Naruse et al., 2020) |
| c.21568C>T | p.R7190* | 10 | (Peng et al., 2018) |
| c.21955C>T | p.Q7319* | 15 | (Duan et al., 2021) |
| c.22835_22836delAA | p.K7612Sfs*28 | 15 | (Naruse et al., 2020) |
| c.20263C>T | p.R6755* | 13 | (Indelicato et al., 2019) |
| c.23237_23238insA | p.L7747Afs*28 | 12 | (Duan et al., 2021) |
| c.23624delT | p.V7875Afs*47 | 24 | (Duan et al., 2021) |
| c.25114C>T | p.R8372* | 27 | (Arias et al., 2019) |
| c.25954C>T | p.R8652* | 18 | (Duan et al., 2021) |

**References:**

Duan, X., Hao, Y., Cao, Z., Zhou, C., and Zhang, J., et al. (2021). Autosomal recessive cerebellar ataxia type 1: Phenotypic and genetic correlation in a cohort of chinese patients with SYNE1 variants. *Cerebellum.* 20, 74-82. doi:10.1007/s12311-020-01186-8.

Holla, V.V., Surisetti, B.K., Prasad, S., and Pal, P.K. (2021). Focal dystonia in a case of SYNE1 spastic-ataxia: Expanding the phenotypic spectrum. *Parkinsonism & Related Disorders.* 87, 22-24. doi: 10.1016/j.parkreldis.2021.04.014.

Indelicato, E., Nachbauer, W., Fauth, C., Krabichler, B., and Schossig, A., et al. (2019). SYNE1-ataxia: Novel genotypic and phenotypic findings. *Parkinsonism & Related Disorders.* 62, 210-214. doi:10.1016/j.parkreldis.2018.12.007.

Kume, K., Morino, H., Komure, O., Matsuda, Y., and Ohsawa, R., et al. (2019). C-terminal mutations in SYNE1 are associated with motor neuron disease in patients with SCAR8. *J. Neurol. Sci.* 402:118-120. doi: 10.1016/j.jns.2019.05.001.

Naruse, H., Ishiura, H., Mitsui, J., Takahashi, Y., and Matsukawa, T., et al. (2020). Juvenile amyotrophic lateral sclerosis with complex phenotypes associated with novel SYNE1 mutations. *Amyotrophic Lateral Sclerosis & Frontotemporal Degeneration*, 1-3. doi:10.1080/21678421.2020.1813312.

Peng, Y., Ye, W., Chen, Z., Peng, H., and Wang, P., et al. (2018). Identifying SYNE1 ataxia with novel mutations in a chinese population. *Frontiers in Neurology.* 9, 1111. doi:10.3389/fneur.2018.01111

Valentina Castillo, J., Catherine Díaz, S., Bustamante, M.L., Ferreira, M.G., and Teive, H.A.G., et al. (2021). Autosomal recessive cerebellar ataxia 1: First case report depicting a variant in SYNE1 gene in a chilean patient. *Cerebellum*. doi:10.1007/s12311-021-01250-x.

Yoshinaga, T., Nakamura, K., Ishikawa, M., Yamaguchi, T., and Takano, K., et al. (2017). A novel frameshift mutation of SYNE1 in a Japanese family with autosomal recessive cerebellar ataxia type 8. *Human Genome Variation.* 4, 17052. doi:10.1038/hgv.2017.52.
